# Molecular Characterisation of the Romanian African Swine Fever Virus p72 and B602L Genetic Markers

**DOI:** 10.3390/vetsci13100997

**Published:** 2026-09-22

**Authors:** Maria Rodica Gurău, Georgeta Ștefan, Alexandru Gligor, Sonia-Lucreția Beșleagă, Florica Bărbuceanu, Stelian Bărăităreanu, Elena Negru, Doru Valentin Hristescu, Vlad Barbu Vuță, Monica Muțiu, Daniel Nicolae Donescu, Ionica Iancu, Viorel Herman, Luminita Costinar, Corina Pascu, Doina Daneș

**Affiliations:** 1 Faculty of Veterinary Medicine, University of Agronomical Sciences and Veterinary Medicine, 050097 Bucharest, Romania; maria.gurau@fmvb.usamv.ro (M.R.G.); sonia-lucretia.besleaga@fmvb.usamv.ro (S.-L.B.); florica.barbuceanu@idah.ro (F.B.); stelian.baraitareanu@fmvb.usamv.ro (S.B.); elena.negru@fmvb.usamv.ro (E.N.); doina.danes@fmvb.usamv.ro (D.D.); 2 Faculty of Veterinary Medicine, University of Life Sciences “King Mihai I”, 300645 Timişoara, Romania; ionica.iancu@usvt.ro (I.I.); viorel.herman@fmvt.ro (V.H.); luminita.costinar@usvt.ro (L.C.); corinapascu@usvt.ro (C.P.); 3 Institute for Diagnosis and Animal Health (IDAH), 050557 Bucharest, Romania; doru.hristescu@idah.ro (D.V.H.); vlad.vuta@idah.ro (V.B.V.); monica.motiu@idah.ro (M.M.); daniel.donescu@idah.ro (D.N.D.); 4 Academy of Romanian Scientists (AOSR), Str. Ilfov, Nr. 3, Sector 5, 50044 Bucharest, Romania

**Keywords:** African swine fever virus, molecular epidemiology, genotype II, p72, B602L, central variable region, wild boar, domestic pig, phylogenetic analysis, genomic surveillance

## Abstract

African swine fever (ASF) remains one of the most devastating transboundary diseases affecting domestic pigs and wild boars worldwide, making molecular epidemiology essential for understanding viral transmission and supporting disease control. This study aimed to characterise African swine fever virus (ASFV) isolates circulating in Romania through comparative analysis of the B646L (p72) gene and the B602L central variable region (CVR). Fifty-four ASFV-positive samples collected from domestic pigs and wild boars were confirmed by real-time PCR and subjected to molecular analysis. Following Sanger sequencing and sequence quality assessment, 32 reliable consensus sequences were retained for B602L and 33 for p72. Sequencing of the p72 gene demonstrated a very high degree of nucleotide conservation, confirming that all successfully sequenced isolates belonged to genotype II. In contrast, the B602L region exhibited limited intra-genotypic variability characterised by scattered single nucleotide polymorphisms and localised insertion/deletion events, providing additional molecular resolution compared to p72, although insufficient for fine-scale epidemiological tracking. No genetic segregation according to host species was observed for either genomic region, and sequences obtained from domestic pigs and wild boar were interspersed in the phylogenetic analyses, demonstrating a high degree of genetic similarity at the investigated loci. These findings highlight the complementary value of p72 and B602L for ASFV molecular characterisation, while also indicating that these partial genomic markers alone cannot resolve transmission pathways or epidemiological links between domestic and wild host populations.

## Simple Summary

African swine fever is a severe viral disease that affects domestic pigs and wild boars and causes major economic losses worldwide. Understanding the virus’ genetic characteristics is important for monitoring its circulation and supporting disease control. This study examined 54 virus-positive samples collected from domestic pigs and wild boars in Romania between 2019 and 2022. Two regions of the viral genetic material were investigated to assess the genetic characteristics and relationships among the viruses. The results showed that the analysed viruses belonged to the same major genetic group and were highly similar, with no clear genetic differences between viruses detected in domestic pigs and wild boars. Although the two investigated genetic regions are useful for routine monitoring and comparison of circulating viruses, they do not provide enough detail to determine precise transmission routes between animals or populations. These findings provide useful baseline information on the genetic characteristics of African swine fever virus circulating in Romania and support the use of more detailed genetic approaches for future outbreak investigations.

## 1. Introduction

African swine fever (ASF) is currently recognized as one of the most important transboundary animal diseases affecting domestic pigs (*Sus scrofa domesticus*) and wild boar (*Sus scrofa*) populations worldwide. The disease is caused by African swine fever virus (ASFV), a large enveloped double-stranded DNA virus, the sole member of the family *Asfarviridae*. Since the introduction of genotype II into Georgia in 2007, ASF has spread rapidly across Europe and Asia, transforming from a geographically contained disease into a major global veterinary concern [1]. The continuing spread of ASFV has generated substantial economic losses through direct mortality, trade restrictions, and costs associated with eradication programs [2]. Furthermore, ASF has had a profound impact on food security and sustainable pig production systems, particularly in regions characterised by small-scale and backyard farming [3,4].

The epidemiology of ASF is exceptionally complex and involves interactions among domestic pigs, wild boar populations, environmental reservoirs, contaminated fomites, and, in some endemic regions, biological vectors such as soft ticks of the genus *Ornithodoros* [5]. Unlike many other viral diseases affecting swine, ASF remains difficult to control because no universally available and fully effective commercial vaccine has been implemented on a global scale [6]. Consequently, surveillance, prompt laboratory diagnosis, early outbreak detection, and strict biosecurity measures are critical for disease prevention and control [7]. In this context, molecular epidemiology has become an essential component of modern ASF surveillance systems, providing valuable information regarding virus introduction events, transmission pathways, and outbreak connectivity [8].

ASFV possesses one of the most complex genomes among animal viruses. The viral genome consists of approximately 170–190 kbp of double-stranded DNA and encodes more than 150 proteins involved in viral replication, host immune modulation, virulence, and particle assembly [2]. Structural studies have revealed that the ASFV virion displays a highly organized multilayered architecture composed of an external envelope, an icosahedral capsid, an internal lipid membrane, and a nucleoprotein core containing the viral genome [9,10]. Numerous viral proteins interact during virion assembly, and alterations affecting these proteins may influence viral replication efficiency, pathogenicity, and epidemiological fitness [11,12].

Among the structural components of ASFV, the major capsid protein p72 represents one of the most important viral proteins. Encoded by the B646L gene, p72 constitutes the predominant structural element of the viral capsid, being directly responsible for the formation of the characteristic icosahedral architecture of the virion. Experimental studies have demonstrated that suppression of p72 expression leads to profound defects in virion morphogenesis. Under restrictive conditions, infected cells accumulate aberrant membrane-associated structures known as “zipper-like structures”, which fail to develop into mature virions. Restoration of p72 expression allows progressive capsid assembly and the generation of infectious viral particles, confirming the essential role of p72 during virus morphogenesis [13].

Beyond its structural importance, p72 has become the principal molecular marker used for ASFV classification and genotyping. Owing to its relatively conserved sequence, partial sequencing of the B646L gene underpins the internationally accepted ASFV genotyping system. Although the original genotype classification was established using the B646L (p72) gene [14], subsequent studies have expanded and refined the phylogenetic framework [15,16]. Sequence analysis of the p72 gene has proven to be a valuable tool for tracing the origin of outbreaks and investigating the evolutionary relationships among circulating ASFV strains [17,18]. Currently, more than twenty ASFV genotypes have been identified based on p72-based phylogenetic analysis [18].

Despite its usefulness for genotype assignment, p72 exhibits relatively limited variability among closely related isolates. Consequently, its discriminatory power may be insufficient when investigating local transmission events involving strains belonging to the same genotype. This limitation has stimulated the search for additional molecular markers capable of providing higher epidemiological resolution [19].

Among these markers, the B602L gene has emerged as one of the most informative genomic targets for fine-scale molecular epidemiology. It encodes the non-structural protein pB602L, which functions as a molecular chaperone involved in the folding, maturation, and stabilization of the major capsid protein p72. Experimental repression of pB602L expression impairs capsid formation, further supporting its essential role as a molecular chaperone during ASFV virion morphogenesis [20].

From a molecular epidemiology perspective, the significance of B602L derives primarily from the presence of highly variable genomic regions, particularly the Central Variable Region (CVR). The central variable region (CVR) of the B602L gene contains tandem amino acid repeat sequences that exhibit substantial genetic variability among ASFV isolates, providing considerably higher discriminatory power than p72-based genotyping alone [21]. Subsequent studies have confirmed the value of B602L-CVR characterisation for outbreak tracing, identification of transmission pathways, and differentiation of closely related ASFV strains circulating within the same genotype [22,23,24,25,26,27].

The complementary nature of p72 and B602L makes their combined analysis particularly valuable in regions where genotype II ASFV predominates and supports their combined use for molecular characterisation of ASFV. In regions dominated by genotype II, p72 (B646L) sequencing provides reliable genotype assignment but has limited discriminatory resolution among closely related isolates, whereas analysis of the B602L central variable region may reveal additional intra-genotypic variation [26,27,28]. Nevertheless, these two markers alone do not provide sufficient resolution for definitive molecular tracing or reconstruction of transmission pathways. Therefore, the genetic relationships inferred from p72 and B602L should be interpreted cautiously and, where possible, complemented by additional molecular markers, whole-genome sequencing, and epidemiological data.

Recent ASF outbreaks in Europe have highlighted the importance of understanding virus circulation at the wildlife–livestock interface. Wild boar populations contribute to the local maintenance and persistence of ASFV in affected areas, whereas human-mediated activities represent important drivers of virus dissemination, particularly over longer distances. These include movements of infected animals, contaminated vehicles and equipment, and the transport or improper disposal of contaminated animal products, by-products, feed, food waste, and other materials. Backyard and small-scale pig holdings may further contribute to disease persistence and transmission where biosecurity is limited [5,29,30]. Molecular characterisation of ASFV isolates from domestic pigs and wild boars can provide complementary information on the genetic relatedness of circulating viruses. However, p72 and B602L alone provide limited discriminatory resolution and should therefore be interpreted together with epidemiological information and, where available, additional molecular markers or whole-genome data [31,32].

In this context, the present study focused on the molecular characterisation of ASFV isolates through comparative analysis of the p72 (B646L) gene and the central variable region (CVR) of B602L. By combining p72, a highly conserved marker used for genotype assignment, with the comparatively more variable B602L-CVR, the study aimed to assess the genetic diversity and relationships among the analysed ASFV isolates and to within our specific cohort. While acknowledging that these partial genomic markers possess limited discriminatory resolution for fine-scale epidemiological tracing, this study aims to describe the baseline genetic features of circulating strains across domestic and wild host populations in the investigated regions.

## 2. Materials and Methods

### 2.1. Materials

The study included samples collected from domestic pigs (*Sus scrofa domesticus*) and wild boars (*Sus scrofa*) as part of the national African swine fever (ASF) surveillance programme and routine diagnostic investigations conducted between 2019 and 2022.

Sampling was performed in accordance with the diagnostic protocols implemented by the Romanian veterinary authorities. Domestic pig samples originated from both commercial and backyard farms, whereas wild boar samples were obtained from hunting grounds, passive surveillance of animals found dead, and organized hunting activities.

A total of 54 ASFV-positive samples were included in the study, comprising 46 domestic pig samples and 8 wild boar samples. The investigated animals originated from multiple localities within different Romanian counties, representing a broad geographical distribution of ASFV-positive cases. Clinical presentation among domestic pigs varied from acute mortality to fever, anorexia, depression, and hyperthermia, whereas wild boar samples originated primarily from hunted animals or carcasses detected during passive surveillance.

Depending on sample availability and carcass condition, biological material consisted of whole blood or pooled tissue samples (comprising spleen, lymph nodes, liver, and lung) routinely collected for ASF laboratory diagnosis, according to the recommendations of the World Organisation for Animal Health (WOAH). The real-time PCR analyses were executed on these pooled tissue homogenates rather than individual organs. Each sample identifier corresponds to a single individual animal. When multiple samples originated from the same locality, the locality name was retained in the identifier and a numerical suffix was added to distinguish individual animals (e.g., ROASFV-SIHLEA1–ROASFV-SIHLEA8). Thus, sequentially numbered identifiers strictly represent distinct, individual host cases sampled within the same geographical location and do not denote repeated or longitudinal sampling of the same animal Table 1, Figure 1.

### 2.2. Nucleic Acid Extraction

Whole-blood and tissue samples were processed (tissue samples were mechanically homogenized prior to nucleic acid extraction, whereas whole-blood samples were processed directly) prior to molecular analysis, and total nucleic acids were extracted using the QIAamp^®^ cador^®^ Pathogen Mini Kit (Qiagen, Hilden, Germany) according to the manufacturer’s instructions. For each extraction, 200 µL of biological sample was mixed with 20 µL Proteinase K and 100 µL VXL buffer, followed by mixing and incubation for 15 min at room temperature. After addition of 350 µL ACB buffer, the lysate was transferred to the purification column and centrifuged at 6010–9391× *g* using an Eppendorf 5424 centrifuge equipped with an FA-45-24-11 fixed-angle rotor (Eppendorf, Hamburg, Germany) for 1 min. The column was subsequently washed with 600 µL AW1 buffer and 600 µL AW2 buffer, with centrifugation after each washing step. A final centrifugation at maximum speed for 2 min was performed to remove residual wash buffer. Purified nucleic acids were eluted in 50 µL AVE buffer following a 1 min incubation at room temperature and centrifugation at maximum speed for 1 min. Extracted DNA was stored at −80 °C until subsequent molecular analysis.

### 2.3. ASFV Detection by Real-Time PCR

Following nucleic acid extraction, all samples were tested for ASFV DNA by real-time PCR using the African Swine Fever Virus Monodose dtec-qPCR kit (Genetic PCR Solutions™, Orihuela, Spain). Amplification was performed on a Rotor-Gene Q 5plex HRM real-time PCR platform (Qiagen, Hilden, Germany), according to the manufacturer’s recommendations.

For each reaction, 13 µL of DNase-free water supplied with the kit and 7 µL of extracted DNA were added directly to the reaction tube, resulting in a final reaction volume of 20 µL. All samples were analysed in duplicate. To ensure assay workflow reliability, each real-time PCR amplification run incorporated thoroughly validated positive and negative amplification controls to verify master mix functionality and monitor for potential cross-contamination. Crucially, the commercial assay features a multiplexed internal positive control (IPC) serving as an internal/inhibitory control to monitor nucleic acid extraction efficiency and actively screen for PCR enzymatic inhibition. ASFV-specific amplification was monitored in the FAM channel, whereas the internal control was monitored in the HEX channel.

The thermal cycling protocol consisted of an initial activation step at 95 °C for 2 min, followed by 40 cycles of denaturation at 95 °C for 5 s and annealing/extension at 60 °C for 20 s. Fluorescence acquisition was performed during the annealing/extension step.

Cycle threshold (Ct) values were determined using the Rotor-Gene Q software (v2.3). All samples included in the present study tested positive for ASFV DNA, with Ct values not exceeding 26, and were subsequently subjected to conventional PCR amplification of the p72 (B646L) and B602L genomic regions for sequencing and molecular characterisation.

### 2.4. Investigation of Genetic Determinants

#### 2.4.1. Selection of Genetic Targets: p72 and B602L

Two genomic regions were selected for molecular characterisation: the B646L (p72) gene and the central variable region (CVR) of the B602L gene. The p72 gene is the internationally accepted marker for ASFV genotyping because of its high conservation and phylogenetic value, whereas the B602L CVR exhibits greater sequence variability, providing higher discriminatory power for differentiating closely related genotype II isolates and investigating epidemiological relationships [19].

#### 2.4.2. Conventional PCR Amplification Method

Conventional PCR amplification of the p72 (B646L) gene and the central variable region (CVR) of the B602L gene was performed using Platinum™ II Hot-Start Green PCR Master Mix (2X) (Invitrogen™, Thermo Fisher Scientific, Waltham, MA, USA). The master mix contains Platinum™ II Taq Hot-Start DNA Polymerase, Platinum™ II PCR buffer, dNTPs, and tracking dyes suitable for direct loading of amplification products onto agarose gels.

PCR reactions were prepared in a final volume of 25 µL, containing 12.5 µL Platinum™ II Hot-Start Green PCR Master Mix (2X), 0.5 µL forward primer (10 µM), 0.5 µL reverse primer (10 µM), 2 µL extracted DNA, and 9.5 µL nuclease-free water. This resulted in a final primer concentration of 0.2 µM for each primer.

Temperature protocol amplification was performed with an initial denaturation step at 95 °C for 10 min, followed by 40 cycles consisting of denaturation at 95 °C for 30 s, primer annealing for p72 at 60 °C for 60 s and for B602L 65 °C for 60 s, and extension at 72 °C for 90 s. A final extension step was performed at 72 °C for 10 min, followed by holding at 4 °C.

PCR products were evaluated by 1.5% agarose gel electrophoresis and visualized under UV illumination. The expected amplicon sizes were approximately 478 bp for the p72 (B646L) fragment and 665 bp for the B602L central variable region (CVR). A 100 bp DNA molecular weight marker was included to estimate amplicon size (Table 2, Table 3).

Amplicons displaying a single, well-defined band of the expected size were selected for bidirectional Sanger sequencing.

#### 2.4.3. Sanger Sequencing

PCR products were sequenced by the Sanger sequencing method using the same primers employed for conventional amplification. Sequencing reactions were performed by a commercial sequencing service (Antisel/CEMIA), where enzymatic or column-based PCR product purification was systematically executed prior to bidirectional Sanger sequencing. Forward and reverse chromatograms were assembled, manually inspected, and edited using BioEdit software (v7.2.5). Electropherograms were audited based on objective quality criteria, including peak resolution symmetry, signal intensity, and the absence of overlapping peaks or high background noise.

A consensus sequence was retained for subsequent analysis only when the forward and reverse reads showed sufficient sequence quality and absolute concordance to allow reliable consensus reconstruction. Chromatograms displaying poorly resolved or ambiguous signals that precluded definitive consensus calling were systematically excluded from downstream analysis. Consequently, 32 high-quality consensus sequences were retained for B602L and 33 for p72 from the initial set of 54 ASFV-positive samples. Crucially, sample attrition was driven exclusively by these stringent sequence quality thresholds; an audit confirmed that success or failure in consensus generation occurred independently of host species, collection year, geographical origin, or baseline real-time PCR Ct values. Consensus nucleotide sequences were translated into amino acid sequences to identify nucleotide substitutions and amino acid polymorphisms.

#### 2.4.4. Genetic Relationship Among ASFV Isolates

Consensus sequences generated in this study were aligned with representative ASFV reference sequences retrieved from GenBank. Multiple sequence alignments were initially performed using BioEdit/ClustalW and subsequently subjected to manual inspection. Nucleotide identity, amino acid substitutions, and sequence polymorphisms were assessed for the p72 and B602L regions.

Phylogenetic reconstruction was performed using the Phylogeny.fr platform, using the Maximum-Likelihood (ML) method implemented in PhyML (v3.0). The analysis was conducted using the automated configuration for nucleotide substitution model selection, with four substitution rate categories; the gamma distribution shape parameter and the proportion of invariant sites were estimated directly from the empirical dataset. Gap-containing positions were excluded from phylogenetic inference, and poorly aligned or ambiguous regions were removed during alignment curation. Statistical branch support was assessed using the approximate likelihood-ratio test (aLRT) implemented in PhyML, which provides a computationally robust alternative to traditional bootstrapping; therefore, conventional bootstrap resampling was not performed and bootstrap replicate numbers are not applicable. The resulting phylogenetic tree was visualised using TreeDyn.

Reference sequences were retrieved from GenBank and selected based on the availability of the corresponding genomic region, compatibility with the analysed sequence fragment, and their relevance for comparison with the ASFV sequences investigated in the present study.

Because the analysed p72 fragment showed extremely limited sequence variation among the Romanian samples, it did not provide sufficient resolution for an informative phylogenetic reconstruction at the intra-genotypic level. Therefore, p72 was used primarily for genotype assignment, whereas phylogenetic reconstruction was focused on the more variable B602L region.

## 3. Results

### 3.1. Detection of ASFV by Real-Time PCR

A total of 54 samples originating from domestic pigs and wild boars were analysed by real-time PCR for the detection of African swine fever virus (ASFV) DNA. All investigated samples yielded positive amplification results, confirming the presence of ASFV within the study population.

The Ct values obtained ranged from 16.22 to 26.00 cycles. The lowest Ct values were recorded for samples ROASFV-SIHLEA2 (Ct = 16.22) and ROASFV-SIHLEA7 (Ct = 16.40), indicating a high concentration of viral genetic material. Similarly, samples ROASFV-PUFESTI1, ROASFVPUFESTI-4, ROASFV-SIHLEA1, ROASFV-STRAOANE2 and ROASFV-SIHLEA7 presented Ct values below 18 cycles. In contrast, the highest Ct values were observed in samples ROASFV-SIHLEA6 (Ct = 25.00) and ROASFV-MARASESTI1 (Ct = 26.00), suggesting comparatively lower viral loads.

Most quantified samples showed Ct values between 17 and 21 cycles, indicating substantial ASFV genome concentrations within the investigated samples. Positive ASFV real-time PCR results were obtained from both domestic pig and wild boar samples.

Overall, the molecular screening confirmed widespread ASFV infection throughout the investigated population and provided suitable material for subsequent sequencing and comparative genomic analyses. All 54 ASFV-positive samples were subjected to amplification and sequencing procedures for molecular characterisation. Following quality assessment of the forward and reverse sequencing reads, reliable consensus sequences could be reconstructed for 32 samples for the B602L region and 33 samples for the p72 region. The remaining sequencing datasets were excluded because the quality and/or concordance of the forward and reverse reads was insufficient to generate reliable consensus sequences.

Ct values were used descriptively to characterise the molecular detection results and were not interpreted as direct quantitative measures of viral load. Parametric statistical comparisons of Ct variances (such as independent t-tests or ANOVA) across sampling years, host species, or geographical origins were deliberately not executed (Table 4). Because this retrospective diagnostic dataset is fundamentally unbalanced across these categories and lacks standardised source-level metadata regarding critical confounding factors (including the precise clinical or pathological stage of the host at death and the anatomical composition of the organ pools), conducting inferential comparative statistics on template yields would introduce profound confounding bias and lead to epidemiologically misleading correlations.

### 3.2. Molecular Characterisation of the B602L

A total of 32 high-quality consensus sequences corresponding to the B602L genomic region were obtained and included in the comparative analysis. Multiple sequence alignment was performed using the ASFV genotype II reference sequence MH910495.1 (Georgia 2007/1). The resulting alignment comprised 526 nucleotide positions, while the reference sequence spanned 560 bp within the uploaded dataset.

Comparative analysis demonstrated an exceptionally high degree of genetic conservation among the Romanian ASFV isolates. The mean exact pairwise nucleotide identity was 99.75%, with a median value of 100%, whereas pairwise identity ranged from 97.58% to 100%. These findings indicate that the investigated viruses belong to a highly homogeneous genotype II population, differing only by a limited number of nucleotide substitutions and localised insertion/deletion events.

When sequence identity was calculated across the complete multiple alignment, including insertion/deletion events within the central variable region (CVR), the mean alignment identity decreased to 92.38% (median 92.59%; range 74.14–100%). This reduction primarily reflects differences in repeat copy number and gap distribution rather than extensive nucleotide divergence.

A total of 171 variable alignment positions were identified among the 32 Romanian isolates. The observed polymorphisms were concentrated within discrete regions corresponding to the CVR tandem repeat area, whereas the remaining flanking portions of the fragment remained highly conserved. Across the global alignment, the analysed sequences resolved into three distinct groups of identical B602L sequence haplotypes (as detailed in Section 4.1), indicating that only a limited number of closely related molecular variants are actively circulating within the investigated Romanian ASFV population.

Overall, the multiple sequence alignment confirmed that the Romanian genotype II isolates exhibit remarkable genetic homogeneity, with sequence diversity being driven predominantly by localised insertion/deletion polymorphisms within the CVR rather than by widespread nucleotide substitutions.

Exact pairwise nucleotide identity among the Romanian isolates was calculated exclusively across homologous aligned nucleotide positions and demonstrated a mean identity of 99.75%, confirming the high genetic stability of the analysed genotype II viruses. In contrast, full-alignment identity values were lower because they incorporated insertion/deletion events associated with the tandem-repeat architecture of the B602L central variable region. Consequently, alignment-based identity scores should not be interpreted as conventional nucleotide identity but rather as a measure of overall alignment similarity that reflects both nucleotide substitutions and structural length polymorphisms. The observed differences therefore predominantly represent variation in repeat organization within the CVR and are consistent with the known molecular evolution of the B602L gene in genotype II ASFV (Table 5).

The amino acid alignment of the B602L region revealed a predominantly conserved sequence pattern among the analysed ASFV isolates, interrupted by several regions characterised by insertions/deletions and localised amino acid variation. The distribution of gaps observed at the protein level corresponded to indel-containing regions already identified in the nucleotide alignment, supporting the presence of genuine length variation within this genomic region rather than translation-related artifacts. Despite these differences, large portions of the analysed fragment remained highly conserved across the isolates. Overall, the amino acid alignment indicates that B602L variability is mainly concentrated within discrete regions, while the remaining sequence retains a comparatively stable structure (Figure 2).

### 3.3. Phylogenetic Analysis Based on B602L Sequences

Phylogenetic reconstruction based on B602L sequences revealed a predominant cluster containing the majority of the analysed isolates. The overall topology of the tree indicated a high degree of genetic relatedness among sequences, consistent with the elevated pairwise identity values observed in the alignment analysis.

Several secondary branches were identified, corresponding to isolates exhibiting minor nucleotide differences. However, these branches remained closely associated with the principal cluster and did not form clearly separated genetic lineages.

Importantly, no segregation according to host species was observed. Sequences obtained from domestic pigs and those originating from wild boars were interspersed throughout the phylogenetic tree and frequently grouped within the same clusters. Identical or nearly identical sequence profiles were identified in isolates originating from both host populations.

The phylogenetic reconstruction therefore demonstrated the circulation of highly related ASFV strains within the investigated area and confirmed the close genetic relationship between isolates detected in domestic and wild suids.

### 3.4. Molecular Characterisation of the p72

Following sequence quality assessment and alignment processing, 33 valid consensus sequences were retained for the comparative analysis of the p72 genomic region.

Comparative analysis of the p72 fragment demonstrated a markedly different pattern from that observed for B602L. Multiple sequence alignment revealed a very high degree of nucleotide conservation across all investigated isolates.

Only a limited number of nucleotide differences were detected throughout the analysed fragment, and the majority of sequences appeared virtually identical. No consistent mutations associated with host species were identified. Sequences obtained from domestic pigs and wild boars displayed extremely high similarity and clustered within the same genetic group.

Comparison with the genotype II reference sequence (MW828868.1) demonstrated a very high degree of nucleotide identity, supporting the classification of all analysed isolates within ASFV genotype II. The observed sequence conservation supports the suitability of p72 as a stable phylogenetic marker for genotype assignment.

The alignment analysis did not reveal evidence of host-associated genetic differentiation. Instead, the results indicated the presence of a common viral lineage circulating among both domestic pigs and wild boars.

The p72 gene is widely recognized as a highly conserved genomic marker primarily used for ASFV genotype assignment rather than for discrimination among closely related genotype II isolates. Therefore, subsequent quantitative analyses of nucleotide variability were performed on the B602L central variable region, which provides substantially greater epidemiological resolution owing to its tandem repeat polymorphisms and localised insertion/deletion events.

The amino acid alignment of the p72 region revealed a very high degree of conservation among the analysed ASFV isolates. Most amino acid positions were identical across all sequences, with only a limited number of localised differences and ambiguous residues. In contrast to the B602L region, no extensive insertion/deletion patterns were observed, and the overall protein sequence architecture remained highly stable across the analysed isolates. No consistent amino acid variation distinguishing isolates obtained from domestic pigs from those originating from wild boars was identified. These findings further support the highly conserved nature of the p72 region among the investigated ASFV isolates.

### 3.5. Comparative Genomic Analysis of p72 and B602L

Comparative evaluation of the target genomic loci revealed sharply contrasting patterns of molecular conservation. The partial p72 (B646L) fragment demonstrated a highly stable architecture characterised by near-perfect nucleotide identity across all sequences, offering minimal discriminatory utility for sub-genotypic differentiation. Conversely, the central variable region (CVR) of the B602L gene exhibited greater genetic heterogeneity driven by single nucleotide polymorphisms and localised insertion/deletion length variants. Consequently, p72 served exclusively for stable macro-level genotype assignment, whereas B602L offered the primary source of intra-genotypic molecular resolution within our specific sample cohort. A qualitative synthesis of these genomic signatures is summarized in Table 6.

Compared with B602L, the p72 amino acid alignment exhibited markedly greater conservation, emphasizing the contrasting molecular characteristics of the two investigated regions: a highly conserved p72 fragment and a comparatively more variable B602L region characterised by localised substitutions and indel-associated variation.

## 4. Discussion

### 4.1. Discussion of B602L Sequencing Results

The sequencing analysis of the B602L central variable region (CVR) revealed a high degree of genetic similarity among all Romanian ASFV isolates included in this study, despite the variability of this genomic region. Multiple sequence alignment identified only a limited number of single nucleotide polymorphisms (SNPs) and small insertion/deletion (indel) events, while the overall sequence conservation remained remarkably high. These findings indicate that all analysed viruses belong to a closely related viral population and support the continued circulation of a genetically homogeneous genotype II ASFV lineage in Romania [32]. This observation is consistent with previous reports demonstrating the remarkable genomic stability of genotype II ASFV following its introduction into the Caucasus in 2007 and its subsequent spread throughout Europe [2,33,34].

Although the B602L gene is considerably more variable than highly conserved genomic regions such as B646L (p72), the variability observed among the Romanian isolates remained relatively limited [35]. Similar results have been described in Poland, Lithuania, Latvia, Estonia, Germany and Italy, where most genotype II isolates differ only by a few nucleotide substitutions or minor changes within the CVR repeat region [36,37,38]. These observations suggest that, despite continuous circulation over several years, genotype II ASFV accumulates mutations at a relatively slow rate, reflecting the evolutionary stability of this large double-stranded DNA virus.

One of the relevant findings of the present study was the absence of clear genetic separation between sequences obtained from domestic pigs and those obtained from wild boar. Sequences originating from both host species were interspersed throughout the alignment and phylogenetic reconstruction, indicating that the analysed viruses were genetically highly similar at the B602L locus and that the detected B602L variants were not structured according to host origin. However, given the limited discriminatory resolution of a single partial genomic marker and the heavily unbalanced sample size between the two host compartments (46 domestic pigs versus 8 wild boars), this topological mixing must not be interpreted as definitive or standalone evidence of continuous bidirectional transmission or equivalent viral fitness. Rather, it indicates that host-associated differentiation was not detectable within the limits of the B602L fragment analysed in this specific sample cohort. Resolving concrete transmission directionality or reservoir dynamics across the livestock–wildlife interface requires comprehensive whole-genome sequencing (WGS) integrated with precise, large-scale spatiotemporal field metadata [1,39].

Across our dataset, the analysed B602L sequences resolved into three distinct groups of identical sequence haplotypes [19,20]. Haplotype Group 1 comprised the vast majority of our sample cohort (including prominent samples such as ROASFV-SIHLEA1 to ROASFV-SIHLEA5 and ROASFV-PUFESTIV1 to ROASFV-PUFESTIV4), grouping both domestic pig and wild boar isolates from different collection years. Haplotype Group 2 and Group 3 captured minor sequence variants characterised by localised microvariation. Crucially, because formal population-genetic metrics (such as Wright’s fixation index F_ST_ or Median-Joining haplotype network reconstruction) were not executed, our observations remain strictly descriptive. The sharing of homologous sequences across hosts within these haplotype groups cannot be used to mathematically demonstrate the total absence of population structuring or host-specific adaptations across the broader, pan-Romanian ASFV population.

The phylogenetic analysis further confirmed that all Romanian isolates clustered within genotype II, together with previously reported European reference strains. The extremely short branch lengths separating individual isolates indicate recent common ancestry and suggest that the investigated outbreaks originated from the continued circulation of a common viral population rather than from multiple independent introductions of genetically divergent strains. Minor sequence differences detected in several isolates most likely represent local microevolution driven by the gradual accumulation of random mutations during sustained virus transmission, a phenomenon previously described for genotype II ASFV circulating in Europe [34,38].

While p72 sequencing provided robust genotype assignment but limited discrimination among the Romanian isolates, analysis of the B602L CVR revealed additional intra-genotypic variation that was not detectable using p72 alone. The nucleotide substitutions and insertion/deletion polymorphisms identified within B602L allowed a finer molecular differentiation of closely related genotype II isolates. However, these variants did not form consistent host-specific clusters, indicating that B602L provided increased molecular resolution without demonstrating distinct domestic pig- and wild boar-associated lineages. From a molecular epidemiological perspective, the contribution of B602L in the present study was therefore to reveal heterogeneity within an otherwise highly homogeneous genotype II population and to assess whether this variation was associated with host origin. No consistent host-associated pattern was identified, suggesting that the B602L variants detected in domestic pigs and wild boars were not structured according to host species [33].

### 4.2. Discussion of p72 Sequencing Results

Sequencing of the B646L (p72) gene demonstrated an exceptionally high degree of nucleotide conservation among all Romanian ASFV isolates included in this study. Multiple sequence alignment revealed only a limited number of sporadic single nucleotide polymorphisms (SNPs), whereas no major insertions, deletions or clustered mutations were identified. The overall sequence identity remained nearly identical to the reference sequence, confirming the highly conserved nature of the p72 gene. These findings are consistent with numerous previous studies reporting that p72 is one of the most genetically stable regions of the ASFV genome and therefore represents the international reference marker for genotype classification [2,40].

The high sequence conservation observed among the Romanian isolates agrees with reports describing the circulation of genotype II ASFV throughout Europe since its introduction into Georgia in 2007. Although ASFV genomes continue to accumulate mutations during long-term circulation, the p72 coding region remains remarkably stable compared with more variable genomic regions such as the B602L central variable region (CVR) or selected intergenic regions [21,25,38]. Consequently, p72 sequencing remains highly suitable for genotype assignment but provides only limited resolution for distinguishing epidemiologically related isolates.

The extremely high nucleotide identity observed among the analysed sequences indicates that they represent a genetically closely related genotype II population at the investigated loci. Nevertheless, the evidence boundary of this multi-locus partial gene analysis must be clearly outlined. Strains belonging to ASFV genotype II circulating across Europe maintain an exceptionally stable genomic backbone at the p72 and B602L loci [2]. Consequently, combining these two markers is fundamentally insufficient to rule out or differentiate between long-term, continuous local microevolution and multiple independent introduction events of genetically highly identical viral clones from neighbouring endemic regions [32]. Distinguishing between local persistence and repeated trans-boundary influxes requires high-resolution genomics, preferably whole-genome data, thoroughly integrated with temporal, geographical, outbreak, and animal movement vectors [32,39,40].

The lack of consistent genetic differences between sequences obtained from wild boar and domestic pigs indicates that the analysed viruses were highly similar at the p72 locus irrespective of host origin. However, because p72 is a highly conserved marker primarily suitable for genotype assignment, the absence of host-associated differentiation cannot be used to infer transmission events, transmission direction, or epidemiological linkage between domestic pigs and wild boar. These findings should therefore be interpreted as evidence of genetic similarity at this genomic region rather than as direct evidence of virus exchange between host populations [2,40].

### 4.3. Comparative Genomic Analysis of the p72 and B602L Fragments

Comparative analysis of the p72 (B646L) and B602L genomic regions highlighted two distinct patterns of genetic variability within the Romanian ASFV isolates. As expected, the p72 gene exhibited a very high level of nucleotide conservation, with only sporadic single nucleotide substitutions and no evidence of hypervariable regions. This finding confirms the suitability of p72 as the reference marker for ASFV genotype assignment and is consistent with previous studies demonstrating its remarkable genetic stability among genotype II isolates circulating in Europe [2].

In contrast, the B602L central variable region (CVR) displayed a greater degree of sequence variability, characterised by scattered SNPs together with small insertion/deletion events. Although the overall genetic diversity remained limited, the observed polymorphisms provided additional resolution for distinguishing closely related isolates that could not be differentiated using the p72 fragment alone. Similar observations have been reported in several European countries, where B602L-CVR analysis has been used as a complementary marker to increase discrimination among closely related genotype II ASFV isolates [37,38]. Nevertheless, because individual genomic markers capture only a small proportion of ASFV genetic diversity, B602L alone cannot reliably resolve all epidemiological relationships. Higher-resolution investigations therefore require the combined analysis of additional variable genomic regions or, preferably, whole-genome sequence data integrated with temporal and geographical information.

The complementary information provided by these two genomic regions illustrates the importance of combining conserved and variable molecular markers in ASFV molecular epidemiology. While p72 confirmed that all Romanian isolates belonged to the same genotype II lineage, B602L revealed subtle intra-genotypic variation consistent with limited local genetic diversification within the analysed genotype II population. Such minor genetic differences are unlikely to affect genotype assignment but can improve outbreak tracing when interpreted together with epidemiological data.

Importantly, neither genomic region revealed consistent host-associated genetic differentiation. Viruses isolated from domestic pigs and wild boars remained genetically indistinguishable within the conserved p72 fragment and showed only minor sequence variation within B602L, without forming host-specific clusters. These findings support the hypothesis that both host species participate in the maintenance of a common viral population, with continuous transmission occurring between the domestic and wild boar epidemiological cycles [2,40].

Taken together, the comparative genomic analysis demonstrates that Romanian ASFV isolates are characterised by a highly conserved genomic backbone represented by the p72 gene, combined with limited but epidemiologically informative variation within the B602L region. This integrated approach improves the molecular characterisation of circulating ASFV strains and provides valuable information for surveillance, outbreak investigation and reconstruction of transmission pathways.

This strong conservation is consistent with the established role of the B646L gene as a robust marker for ASFV genotype assignment, while also explaining its limited discriminatory capacity for differentiating closely related viruses circulating within the same genotype. In contrast, the B602L amino acid alignment showed greater heterogeneity, including localised amino acid differences and insertion/deletion patterns concentrated within specific regions. Importantly, this variability was not uniformly distributed throughout the analysed B602L fragment, as substantial portions of the sequence remained conserved. The concordance between nucleotide-level indels and the corresponding gaps observed in the amino acid alignment suggests that at least part of the detected B602L diversity is retained after translation and may therefore reflect genuine structural variation among the investigated viral sequences. Nevertheless, neither the p72 nor the B602L amino acid patterns provided evidence of consistent differentiation between viruses detected in domestic pigs and wild boars, supporting the hypothesis of closely related ASFV variants circulating across the domestic–sylvatic interface.

Several limitations should be considered when interpreting the present findings. The samples originated from routine diagnostic and surveillance activities rather than from a prospectively balanced sampling design, resulting in unequal representation across years, geographical areas, and host categories. This sampling structure limits the generalisability of the observed absence of host-associated genetic segregation. Furthermore, no formal population-genetic differentiation analysis or haplotype network analysis was performed; consequently, the distribution of p72 and B602L variants according to host origin should be regarded as descriptive rather than as statistical evidence for the absence of population differentiation. Finally, although Ct values were available for the ASFV-positive samples, statistical comparisons according to year, host, or geographical origin were not performed because potentially relevant confounding factors, including biological sample type and clinical or pathological stage, were not uniformly available. Future studies based on balanced sampling, standardised biological material, whole-genome sequencing, and appropriate multivariable statistical approaches would allow these relationships to be investigated more robustly.

### 4.4. Phylogenetic Analysis

Phylogenetic reconstruction based on the partial B602L nucleotide sequences demonstrated that all Romanian ASFV isolates clustered within the genotype II lineage, together with the Georgia 2007/1 reference strain (MH910495.1) and other representative Eurasian genotype II isolates included in the analysis (Figure 3). No Romanian isolate clustered outside the genotype II clade, confirming the absence of genetically divergent lineages within the analysed dataset.

The B602L phylogenetic analysis showed that the analysed sequences were genetically closely related, with limited differentiation within the overall cluster. Several internal branches displayed relatively low support values, indicating that the corresponding sub-clusters were not robustly resolved. Therefore, the internal topology of these weakly supported branches was not considered sufficient for defining distinct genetic groups.

The B602L central variable region (CVR) showed greater sequence variability than the p72 region (Figure 4, Figure 5). However, the differences detected among the Romanian isolates were relatively limited and did not result in clearly separated phylogenetic groups. Moreover, the observed variation did not produce consistent clustering according to host origin, as sequences obtained from domestic pigs and wild boars remained interspersed within the phylogenetic analysis.

The B602L phylogenetic reconstruction should also be interpreted in light of the relatively low support values observed for some internal branches. The high degree of sequence similarity and small genetic distances among the analysed viruses resulted in limited resolution of several internal relationships. Consequently, weakly supported sub-clusters cannot be considered robustly differentiated lineages and should not be assigned specific epidemiological significance. The tree therefore primarily illustrates the overall genetic relatedness and limited intra-genotypic variation in the analysed B602L sequences rather than providing a robust reconstruction of fine-scale transmission or evolutionary relationships.

## 5. Conclusions

The present study provides a molecular characterisation of two primary genomic markers, p72 (B646L) and B602L, in African swine fever virus (ASFV) isolates collected from domestic pigs and wild boars in Romania. Sequencing of the highly conserved p72 region confirmed that all successfully analysed isolates belong to genotype II, demonstrating high genetic stability that supports its utility for broad macro-level classification. In contrast, the B602L central variable region (CVR) revealed limited intra-genotypic variation characterised by single nucleotide polymorphisms and localised insertion/deletion events, providing additional structural resolution suitable for describing sub-genotypic variation within the investigated cohort.

No host-associated genetic segregation or distinct lineages were detected at either of the investigated loci, and homologous sequences were shared across domestic and wild hosts within the identified haplotype groups. However, given the limited discriminatory resolution of these partial genomic markers and the unbalanced spatiotemporal design of this retrospective surveillance cohort, these descriptive findings cannot be extrapolated as standalone evidence of continuous bidirectional transmission or equivalent viral fitness at the population level.

Consequently, partial p72 and B602L sequencing remains a robust tool for routine genotype monitoring, but it is fundamentally insufficient to differentiate between localised endemic persistence and multiple, independent trans-boundary introduction events of highly uniform genotype II clones. Future high-resolution genomic surveillance programs must integrate whole-genome sequencing (WGS) with comprehensive, standardised spatiotemporal field metadata to precisely reconstruct transmission pathways and support evidence-based African swine fever control measures across Eastern Europe.

## Figures and Tables

**Figure 1:**
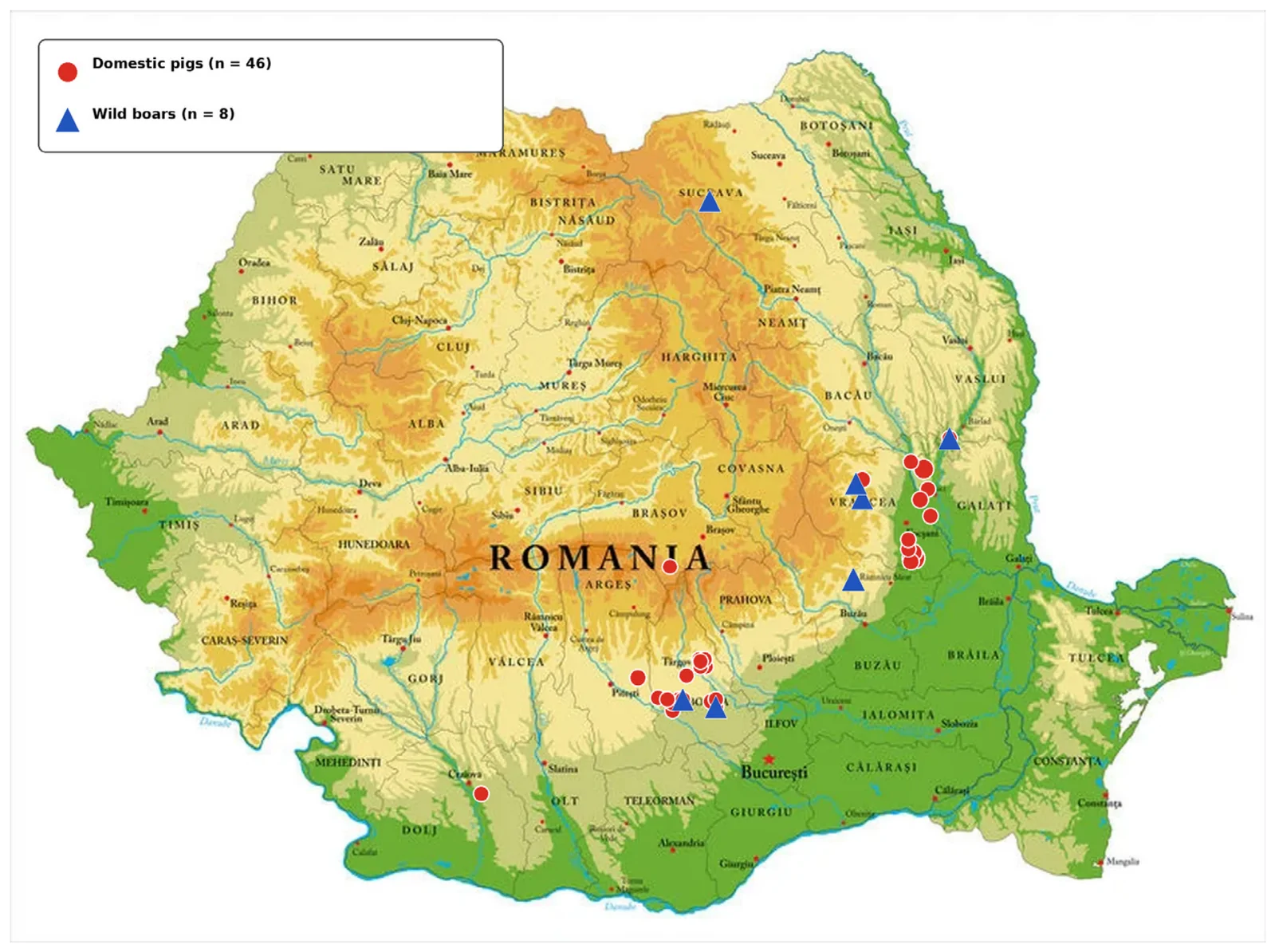
Spatial distribution of sampling locations for ASFV-positive domestic pigs and wild boars in Romania, (2019–2022).

**Figure 2:**
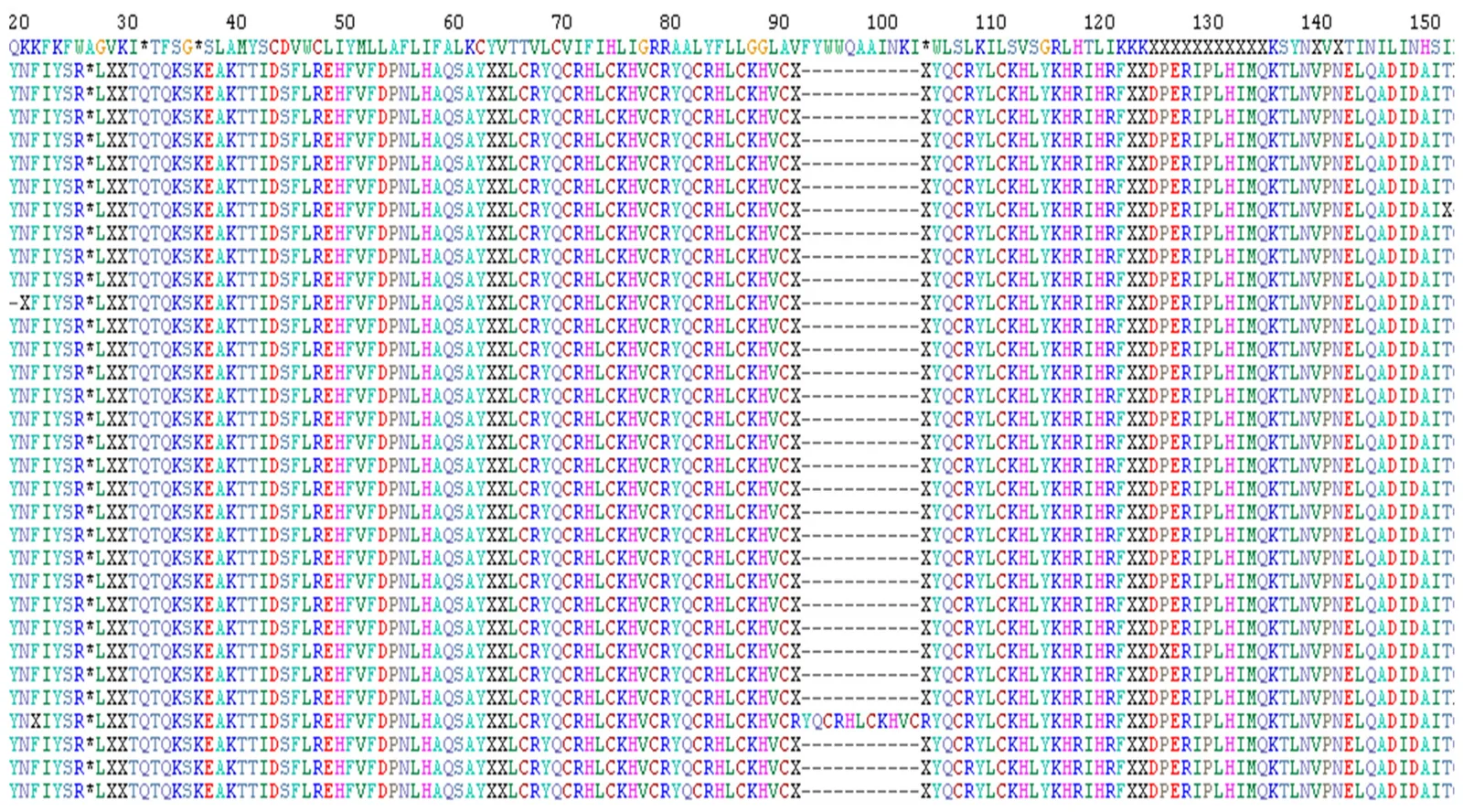
Multiple amino acid sequence alignment of the ASFV B602L region from the analysed Romanian isolates. Colours distinguish amino acid residues according to the BioEdit display colour scheme and are provided solely to facilitate visual interpretation; hyphens indicate alignment gaps.

**Figure 3:**
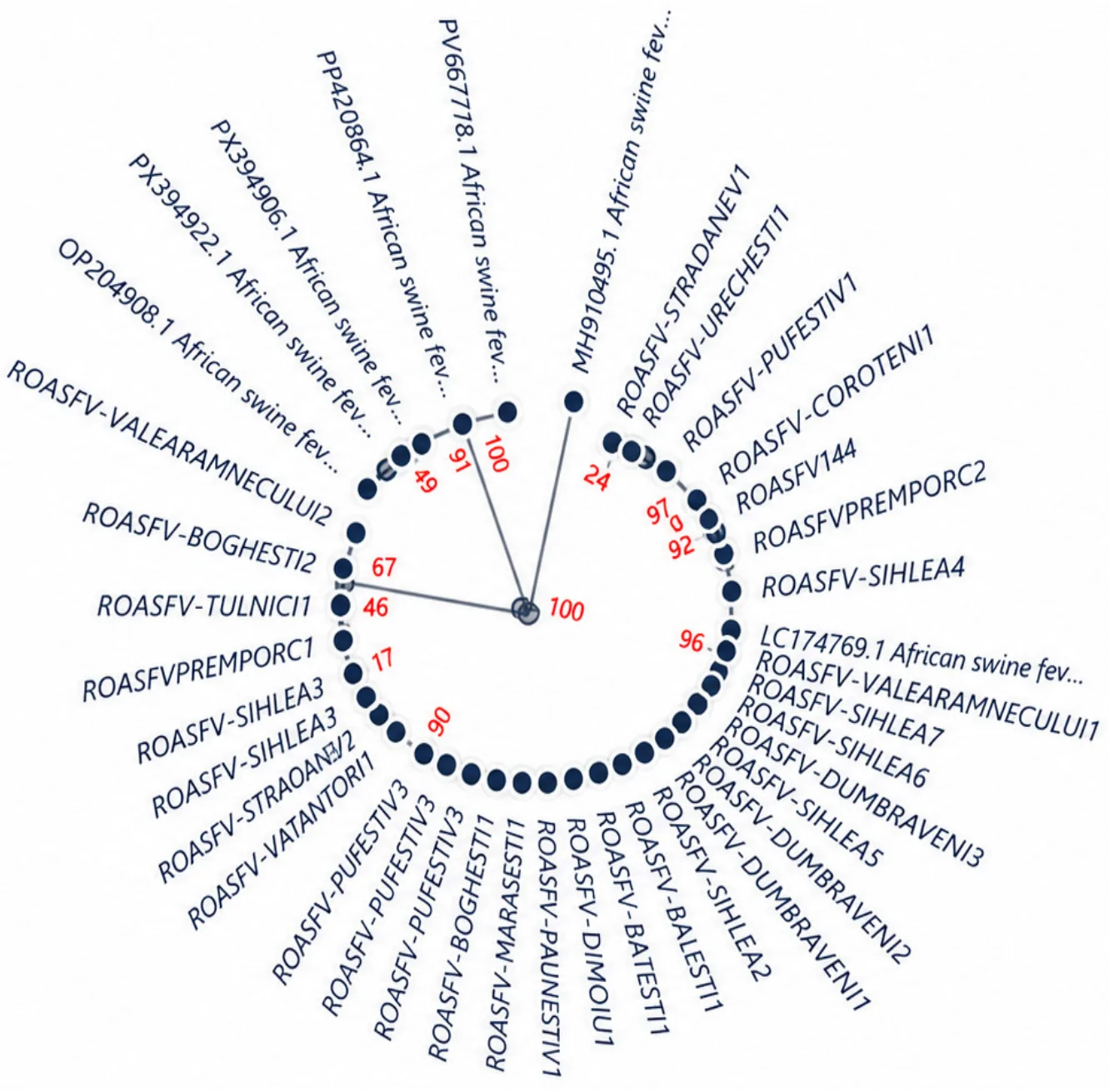
Phylogenetic tree based on B602L nucleotide sequences. Numbers at the internal nodes indicate approximate likelihood-ratio test (aLRT) branch support values (%), calculated using PhyML.

**Figure 4:**
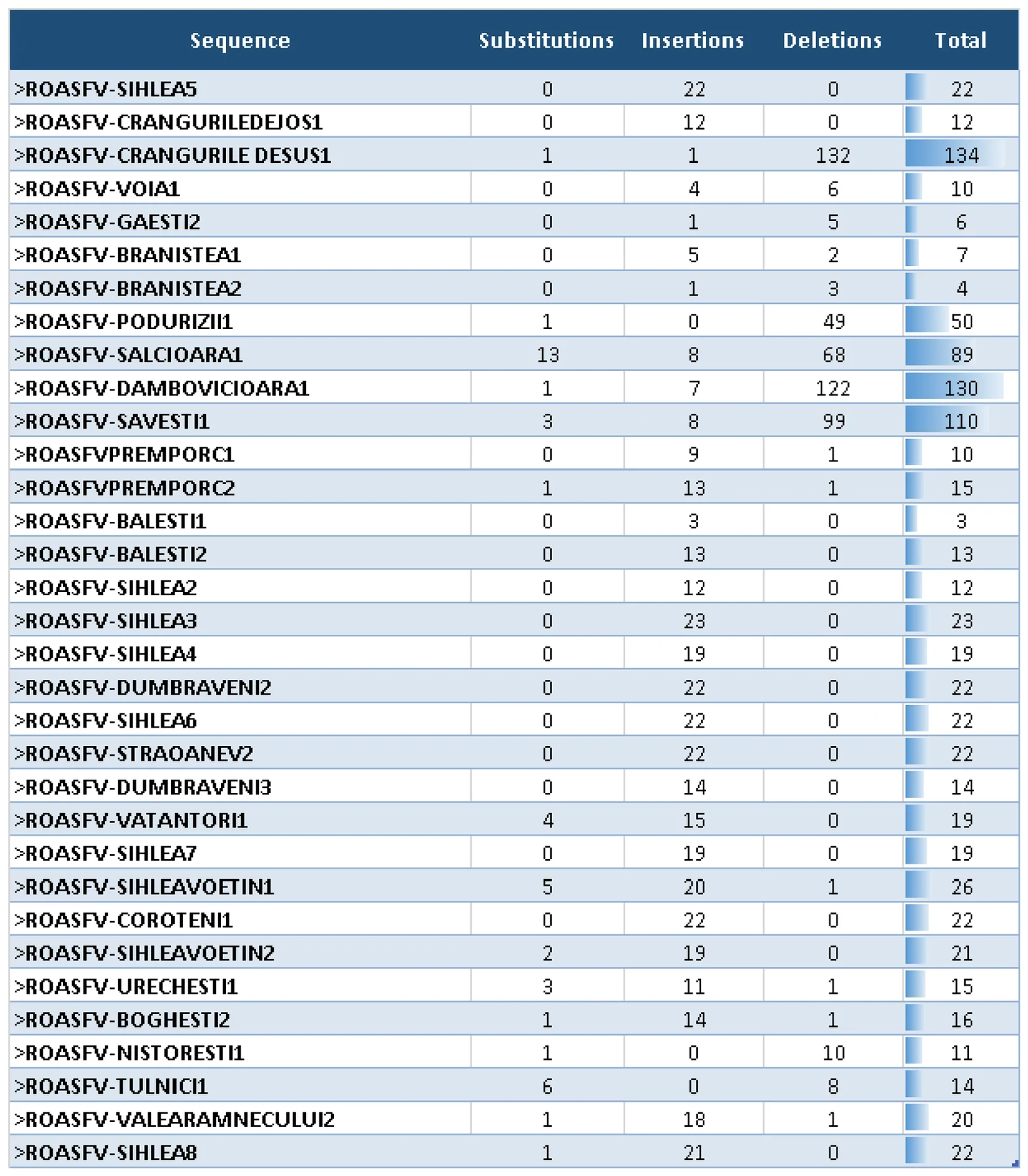
Distribution of nucleotide polymorphisms identified in the p72 gene of Romanian ASFV isolates relative to the genotype II reference sequence (MW828868.1).

**Figure 5:**
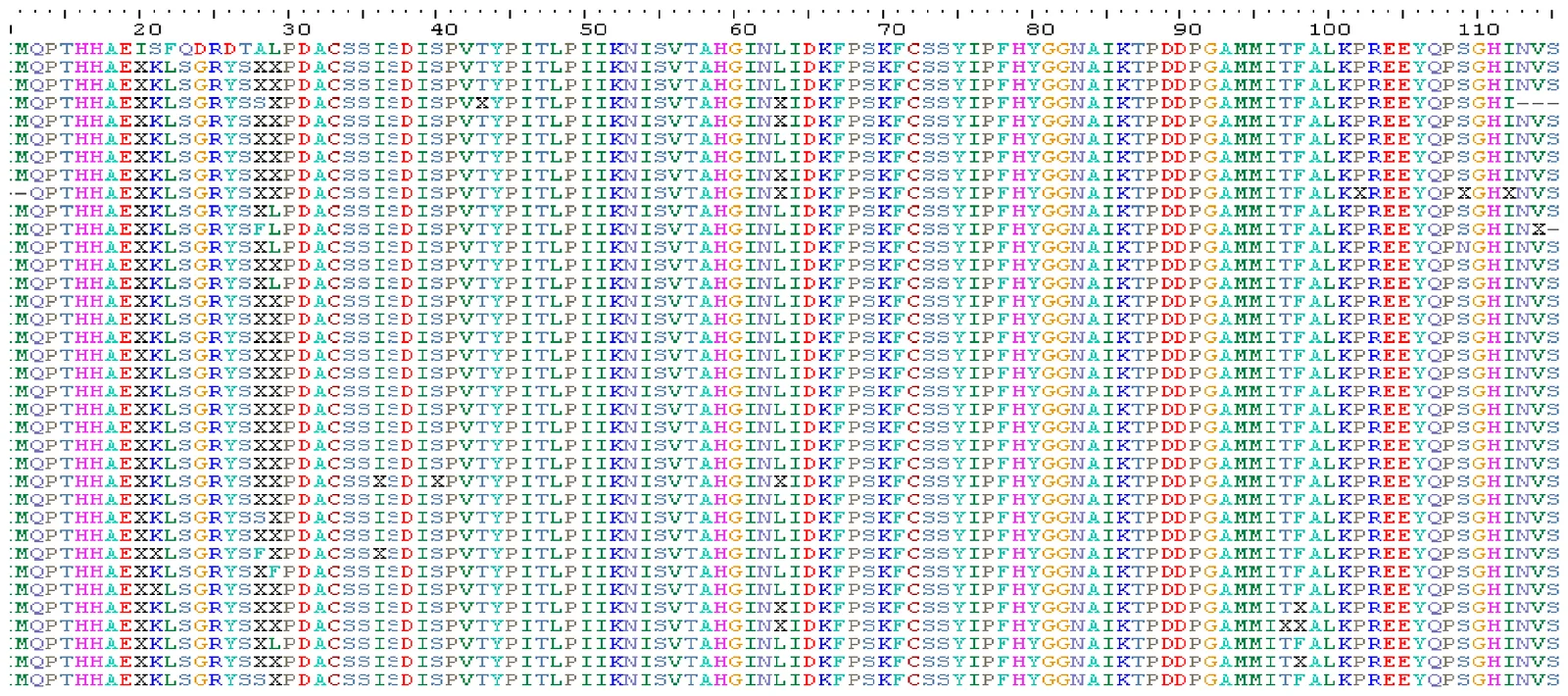
Multiple amino acid sequence alignment of the ASFV p72 region from the analysed Romanian isolates. Colours distinguish amino acid residues according to the BioEdit display colour scheme and are provided solely to facilitate visual interpretation; hyphens indicate alignment gaps.

**Table 1:** Epidemiological metadata and real-time PCR cycle threshold (Ct) values of the 54 Romanian African swine fever virus (ASFV) samples included in the study.

NR.	SAMPLE *	DOMESTIC/BOAR	YEAR	LOCALITY	COUNTY	Ct
1.	>ROASFV-PUFESTIV1	Domestic	2022	Pufesti	Vrancea	17.54
2.	>ROASFV-PUFESTIV2	Domestic	2022	Pufesti	Vrancea	18.48
3.	>ROASFV-PUFESTIV3	Domestic	2022	Pufesti	Vrancea	17.72
4.	>ROASFV-PUFESTIV4	Domestic	2022	Pufesti	Vrancea	17.34
5.	>ROASFV-BOGHESTI1	Domestic	2022	Boghesti	Vrancea	25.00
6.	>ROASFV-MARASESTI1	Domestic	2022	Marasesti	Vrancea	26.00
7.	>ROASFV-STRAOANEV1	Domestic	2022	Straoane	Vrancea	19.13
8.	>ROASFV-PAUNESTIV1	Domestic	2022	Paunesti	Vrancea	18.15
9.	>ROASFV-DIMOIU1	Domestic	2022	Dimoiu	Dambovita	18.33
10.	>ROASFVNISIPURI1	Domestic	2022	Nisipuri	Dolj	22.42
11.	>ROASFV-ULMI1	Domestic	2022	Ulmi	Dambovita	19.09
12.	>ROASFV-MATRACA1	Domestic	2022	Matraca	Dambovita	21.01
13.	>ROASFV-VALEAVOIEVOZI1	Domestic	2022	Valea Voievozilor	Dambovita	17.42
14.	>ROASFV-TARGOVISTE1	Domestic	2022	Targoviste	Dambovita	16.22
15.	>ROASFV-GAESTI1	Boar	2022	Gaesti	Dambovita	19.09
16.	>ROASFV-PATROAIAVALE1	Domestic	2022	Patroaia Vale	Dambovita	18.19
17.	>ROASFV-RATESTI1	Domestic	2022	Ratesti	Arges	19.43
18.	>ROASFV-CRANGURILEDEJOS1	Domestic	2022	Crangurile de jos	Dambovita	18.59
19.	>ROASFV-CRANGURILE DESUS1	Domestic	2022	Crangurile de sus	Dambovita	18.66
20.	>ROASFV-VOIA1	Domestic	2022	Voia	Dambovita	17.05
21.	>ROASFV-GAESTI2	Domestic	2022	Gaesti	Dambovita	20.10
22.	>ROASFV-BRANISTEA1	Boar	2022	Branistea	Dambovita	22.05
23.	>ROASFV-BRANISTEA2	Domestic	2022	Branistea	Dambovita	18.21
24.	>ROASFV-PODURIZII1	Domestic	2022	Podu Rizii	Dambovita	18.88
25.	>ROASFV-SALCIOARA1	Domestic	2022	Salcioara	Dambovita	17.58
26.	>ROASFV-DAMBOVICIOARA1	Domestic	2022	Dambovicioara	Arges	16.40
27.	>ROASFV-SAVESTI1	Domestic	2022	Savesti	Dambovita	18.35
28.	>ROASFVPREMPORC1	Domestic	2021	Golesti	Arges	16.95
29.	>ROASFVPREMPORC2	Domestic	2021	Golesti	Arges	25.88
30.	>ROASFV-BALESTI1	Domestic	2021	Balesti	Vrancea	22.09
31.	>ROASFV-BALESTI2	Domestic	2021	Balesti	Vrancea	16.72
32.	>ROASFV-SIHLEA1	Domestic	2021	Sihlea	Vrancea	17.21
33.	>ROASFV-SIHLEA2	Domestic	2021	Sihlea	Vrancea	16.94
34.	>ROASFV-SIHLEA3	Domestic	2021	Sihlea	Vrancea	17.89
35.	>ROASFV-SIHLEA4	Domestic	2021	Sihlea	Vrancea	25.47
36.	>ROASFV-DUMBRAVENI1	Domestic	2021	Dumbraveni	Vrancea	18.44
37.	>ROASFV-DUMBRAVENI2	Domestic	2021	Dumbraveni	Vrancea	18.73
38.	>ROASFV-SIHLEA5	Domestic	2021	Sihlea	Vrancea	19.39
39.	>ROASFV-SIHLEA6	Domestic	2021	Sihlea	Vrancea	17.10
40.	>ROASFV-STRAOANEV2	Domestic	2021	Straoane	Vrancea	17.30
41.	>ROASFV-DUMBRAVENI3	Domestic	2021	Dumbraveni	Vrancea	19.65
42.	>ROASFV-VATANTORI1	Domestic	2021	Vanatori	Vrancea	18.13
43.	>ROASFV-SIHLEA7	Domestic	2021	Sihlea	Vrancea	17.25
44.	>ROASFV-SIHLEAVOETIN1	Domestic	2021	Sihlea-Voentin	Vrancea	16.58
45.	>ROASFV-COROTENI1	Domestic	2021	Coronteni	Vrancea	18.99
46.	>ROASFV-SIHLEAVOETIN2	Domestic	2021	Sihlea-Voentin	Vrancea	19.06
47.	>ROASFV-URECHESTI1	Domestic	2021	Urechesti	Vrancea	16.84
48.	>ROASFV-BOGHESTI2	Boar	2020	Boghesti	Vrancea	25.92
49.	>ROASFV-NISTORESTI1	Boar	2021	Nistoresti	Vrancea	24.32
50.	>ROASFV-OSVALEAPUTNEI1	Boar	2021	Valea Putnei	Suceava	21.41
51.	>ROASFV-VALEARAMNECULUI1	Boar	2019	Valea Ramnecului	Buzau	16.45
52.	>ROASFV-TULNICI1	Boar	2021	Tulnici	Vrancea	17.25
53.	>ROASFV-VALEARAMNECULUI2	Boar	2020	Valea Ramnecului	Buzau	16.71
54.	>ROASFV-SIHLEA8	Domestic	2021	Sihlea	Vrancea	16.54

* Sample identifiers sharing the same locality name and differing by numerical suffix correspond to different individual animals sampled within that locality. Ct values represent molecular detection results obtained from the pooled organ matrices.

**Table 2:** Primer sequences used for conventional PCR amplification of the B602L central variable region (CVR), including the corresponding expected amplicon size.

Primer	Sequence	Expected Amplicon Size (bp)
CVR1	5ACTTTGAAACAGGAAAC (AT) AATGATG3	665 bp
CVR2	5ATATTTTGTAATATGTGGGCTGCTG3	

**Table 3:** Primer sequences used for conventional PCR amplification of the B646L (p72) genomic region, including the corresponding expected amplicon size.

Primer	Sequence	Expected Amplicon Size (bp)
p72-D	5GGCACAAGTTCGGACATGT3	478 bp
p72-U	5GTACTGTAACGCAGCACAG3	

**Table 4:** Real-time PCR detection of ASFV in samples collected from domestic pigs and wild boars.

Parameter	Value
Total samples analysed	54
ASFV-positive samples	54 (100%)
Minimum Ct ^1^ value	16.22
Maximum Ct value	26.00
Mean Ct value	19.28
Median Ct value	18.61
Predominant Ct interval	17–21
Sample with lowest Ct value	ROASFV-SIHLEA2 (Ct = 16.22)
Sample with highest Ct value	ROASFV-MARASESTI1 (Ct = 26.00)

^1^ Ct = cycle threshold value obtained by real-time PCR.

**Table 5:** Summary statistics of B602L sequence variability.

Parameter	Value
Total sample sequences analysed	32
Uploaded Georgia reference	MH910495.1
Reference length in uploaded file (bp)	560
Sample alignment length (bp)	526
Mean exact pairwise identity among samples (%)	99.7533
Median exact pairwise identity among samples (%)	100
Exact pairwise identity range among samples (%)	97.5771–100.0000
Mean full-alignment identity including gaps (%)	92.3774
Median full-alignment identity including gaps (%)	92.5856
Full-alignment identity range including gaps (%)	74.1445–100.0000
Variable alignment positions among samples	171
Identical sequence groups	3

**Table 6:** Qualitative comparison of genomic characteristics of p72 and B602L fragments.

Characteristic	p72	B602L
Gene type	Major capsid protein	Variable protein
Degree of conservation	Very high	Low
Genetic variability	Low	High
Number of SNPs	Very low	Increased
Insertions/deletions (indels)	Rare/absent	Frequent
Hypervariable regions	Absent	Present
Main application	ASFV genotyping	Intra-genotypic discrimination
Wild boar versus domestic pig differentiation	Not observed	Limited microvariation
Evolutionary stability	High	Low
Epidemiological value	Genotype identification	Outbreak tracing

## Data Availability

The original contributions presented in this study are included in the article. Further inquiries can be directed to the corresponding authors. The newly generated ASFV p72 and B602L consensus nucleotide sequences have not yet been deposited in GenBank, as they form an institutional part of an ongoing national research project (Project No. ADER 10.1.1, 2024–2026). In accordance with the data management and dissemination schedule mandated by the funding body, the sequences will be formally submitted to GenBank immediately upon the administrative completion of the project phase, and the corresponding public accession numbers will be made available thereafter.

## Abbreviations

The following abbreviations are used in this manuscript:

ASF	African Swine Fever
ASFV	African Swine Fever Virus
bp	Base pairs
CVR	Central Variable Region
Ct	Cycle threshold
DNA	Deoxyribonucleic acid
HR	Hypervariable Region
IDAH	Institute for Diagnosis and Animal Health
PCR	Polymerase Chain Reaction
SNP	Single Nucleotide Polymorphism
